# Effects of Atrial Fibrillation on Heart Failure Outcomes and NT-proBNP Levels in the GUIDE-IT Trial

**DOI:** 10.1016/j.mayocpiqo.2021.02.005

**Published:** 2021-04-08

**Authors:** Fouad Chouairi, Justin Pacor, P. Elliott Miller, Michael A. Fuery, Cesar Caraballo, Sounok Sen, Eric S. Leifer, G. Michael Felker, Mona Fiuzat, Christopher M. O’Connor, James L. Januzzi, Daniel J. Friedman, Nihar R. Desai, Tariq Ahmad, James V. Freeman

**Affiliations:** a Section of Cardiovascular Medicine, Yale University, New Haven, CT; b Center for Outcomes Research & Evaluation, Yale University, New Haven, CT; c Department of Internal Medicine, Yale University, New Haven, CT; d Yale School of Medicine, Yale University, New Haven, CT; e Division of Cardiovascular Sciences, National Heart, Lung, and Blood Institute, Bethesda, MD; f Duke Clinical Research Institute, Durham, NC; g Inova Heart and Vascular Institute, Falls Church, VA; h Massachusetts General Hospital, Boston

**Keywords:** AF, atrial fibrillation, CV, cardiovascular, GDMT, guideline-directed medical therapy, GUIDE-IT, Guiding Evidence-Based Therapy Using Biomarker-Intensified Treatment in Heart Failure trial, HF, heart failure, HFrEF, heart failure with reduced ejection fraction, KCCQ, Kansas City Cardiomyopathy Questionnaire, NT-proBNP, N-terminal pro–B type natriuretic peptide, RCT, randomized controlled trial

## Abstract

**Objective:**

To evaluate effects of atrial fibrillation (AF) on cardiac biomarkers and outcomes in a trial population of patients with heart failure (HF) with reduced ejection fraction treated with optimal guideline-directed medical therapy.

**Methods:**

We performed a secondary analysis of 894 patients in the Guiding Evidence-Based Therapy Using Biomarker-Intensified Treatment in Heart Failure (GUIDE-IT) trial (January 2013–July 2016). Patients were stratified by AF status and compared with regard to guideline-directed medical therapy use, longitudinal levels of N-terminal pro–B type natriuretic peptide (NT-proBNP), and outcomes including HF hospitalization and mortality.

**Results:**

After adjustment, AF was associated with a significant increase in the risk of HF hospitalization or cardiovascular death (hazard ratio, 1.28; 95% CI, 1.02 to 1.61; *P*=0.04) and HF hospitalization (hazard ratio, 1.31; 95% CI, 1.02 to 1.68; *P*=.03) but with no difference in mortality during a median 15 months of follow-up. There were no significant differences in medication treatment between those with and those without AF. At 90 days, a higher proportion of patients with AF (89.4% vs 81.5%; *P*=.002) had an NT-proBNP level above 1000 pg/mL (to convert NT-proBNP values to pmol/L, multiply by 0.1182), and AF patients had higher NT-proBNP levels at all time points through 2 years of follow-up.

**Conclusion:**

Among patients with HF with reduced ejection fraction, prevalent AF was associated with higher NT-proBNP concentrations through 2 years of follow-up and higher risk for HF hospitalization despite no substantial differences in medical therapy.

The Guiding Evidence-Based Therapy Using Biomarker-Intensified Treatment in Heart Failure (GUIDE-IT) trial was a randomized controlled trial (RCT) designed to evaluate the efficacy of an N-terminal pro–B type natriuretic peptide (NT-proBNP)–guided treatment plan in patients with clinical heart failure (HF) and a reduced left ventricular ejection fraction below 40%.^1^ Atrial fibrillation (AF) is the most common arrhythmia in patients with HF with reduced ejection fraction (HFrEF), occurring in 15% to 35% of these patients.^2^, ^3^, ^4^, ^5^, ^6^ AF has been associated with a poorer prognosis in HFrEF, including a greater risk of death, but there are limited data of its effects in a well-treated contemporary trial population.^7^, ^8^, ^9^, ^10^ Biomarkers such as natriuretic peptides, specifically NT-proBNP, are known to be powerful predictors of adverse outcomes in patients with chronic HFrEF,^11^ and patients with HFrEF and AF have been shown to have higher NT-proBNP levels than those without AF.^12^ However, data on the magnitude of this difference are limited, and little is known about how levels of this important biomarker change over time with titration of guideline-directed medical therapy (GDMT) for HFrEF with and without AF.

The aim of this study was to investigate the effects of AF on cardiovascular (CV) outcomes in a contemporary trial population of patients with HFrEF treated with a goal of optimal GDMT using data from the GUIDE-IT trial. We further compared NT-proBNP concentrations longitudinally for patients with HFrEF with and without AF to better understand how AF affects levels of this important biomarker over time with titration of GDMT.

## Methods

This study uses the data from the GUIDE-IT trial, the results of which have previously been published.^1^ This trial was a randomized multicenter clinical trial conducted at 45 clinical sites in the United States and Canada. Patients with HFrEF (ejection fraction <40%), NT-proBNP concentration above 2000 pg/mL (to convert NT-proBNP values to pmol/L, multiply by 0.1182) or BNP concentration above 400 pg/mL (to convert BNP values to pmol/L, multiply by 0.289) within the preceding 30 days, or a history of a prior HF event within the preceding 12 months (HF hospitalization or equivalent) were included in the trial. Patients were randomized to either an NT-proBNP–guided strategy or usual care. Those randomized to the guided strategy had GDMT titrated to achieve GDMT targets when possible but with a parallel goal of achieving a target NT-proBNP concentration of less than 1000 pg/mL. Patients randomized to the usual care arm were managed as recommended in HF clinical practice guidelines.^1^^,^^13^ The primary end points were time to first HF hospitalization or CV death, and the trial was stopped because of futility after 894 of the planned 1100 patients had been enrolled. The trial data are publicly available through the Biologic Specimen and Data Repository Information Coordinating Center (BioLINCC). The Yale University Institutional Review Board approved requisition of the data, and waiver of consent was provided to conduct this study.

The Kansas City Cardiomyopathy Questionnaire (KCCQ) was used to assess quality of life. The KCCQ is a 23-question instrument that examines the impact of HF on a patient’s life. This study used the overall summary score as the primary measure. It includes subsection measures of physical limitations, total symptoms, quality of life, and social limitations.^14^^,^^15^ The KCCQ overall summary scores were calculated from subsection scores according to the original methodology used by Green et al.^15^

For this study, participants of the GUIDE-IT trial were stratified into 2 cohorts: those with AF and those without AF. Atrial fibrillation was defined as the presence of AF or atrial flutter in the medical records or on examination during the medical history on enrollment of the patient.^16^^,^^17^ Demographic characteristics, comorbidities, procedures, and baseline characteristics of patients were compared by *t*-tests for continuous variables and *χ*^2^ tests for categorical variables. Per the annotated forms for the GUIDE-IT trial, ablation was characterized as occurring during hospitalizations after the initial patient recruitment and history for the trial. Of note, baseline anticoagulation was defined as anticoagulation by warfarin, direct Xa inhibitor, or direct thrombin inhibitor at initial patient history. The primary outcomes of this study included all-cause death, CV death, first HF hospitalization, and the primary end point of the original trial—HF hospitalization or CV death. For our main outcomes, all-cause death, CV death, non-CV death, HF hospitalization, any hospitalization, and HF hospitalization or CV death, Cox regression was used. For all analyses, there were 2 regressions. The first regression was unadjusted. The second regression model was adjusted for age, sex, diabetes, chronic kidney disease, prior myocardial infarction, prior stroke, ventricular arrhythmia, baseline NT-proBNP concentration, and baseline ejection fraction consistent with the primary analyses for the GUIDE-IT trial and with additions to control for the increased comorbidity burden of patients with AF.^1^ Data were displayed as hazard ratios with 95% CIs for the Cox regressions. The Cox regression analysis was then repeated on the basis of subgroups divided by NT-proBNP concentration lower than 1000 pg/mL and NT-proBNP concentration of 1000 pg/mL and higher. A Kaplan-Meier cumulative event curve for HF hospitalization or CV death was generated with a maximum follow-up of 24 months. Differences in outcomes between patients with and without AF were compared by log-rank tests. The KCCQ overall summary scores were compared at baseline, 3 months, 6 months, 12 months, and 24 months by Mann-Whitney *U* tests. All statistics were done using SPSS 26 software (IBM Corp). The threshold for significance was a 2-sided *P* value of less than .05.

## Results

A total of 358 patients (40.0%) had a history of AF. Table 1 details baseline characteristics categorized as a function of AF. Patients with AF were significantly older, less often female, and more frequently white; they also more commonly had several comorbidities, including kidney disease, sleep apnea, myocardial infarction, ventricular fibrillation or tachycardia, and prior coronary artery bypass graft surgery. Patients with AF generally had higher New York Heart Association class, and fewer AF patients were taking an angiotensin-converting enzyme inhibitor at baseline. These patients also had higher baseline serum creatinine and blood urea nitrogen concentrations.

**Table 1:** Baseline Characteristics of Patients With HFrEF Stratified by Baseline AF in the GUIDE-IT Trial^a^^,^^b^^,^^c^

Characteristics	No AF	AF	*P* value
Overall	535 (59.9)	358 (40.0)	
NT-proBNP–guided arm	281 (52.5)	161 (45.0)	.08
Age (y)	58.4 (14.5)	66.0 (11.5)	**<.001**
Baseline BMI (kg/m^2^)	30.5 (8.8)	29.9 (7.1)	.34
Female	185 (34.7)	98 (27.5)	**.02**
Country			.20
United States	457 (85.7)	294 (82.6)	
Canada	76 (14.3)	62 (17.4)	
Race			
White	259 (48.6)	230 (64.6)	**<.001**
Black	224 (42.0)	98 (27.5)	**<.001**
Asian	14 (2.6)	13 (3.7)	.38
Other	24 (4.5)	12 (3.4)	.40
Comorbidities			
Ischemic heart disease	243 (45.6)	203 (57.0)	**.001**
Diabetes mellitus	234 (43.7)	176 (49.2)	.11
COPD	104 (19.4)	89 (24.9)	.05
Kidney disease	168 (31.4)	162 (45.3)	**<.001**
ICD/CRT	188 (35.1)	207 (57.8)	**<.001**
Sleep apnea	103 (19.3)	98 (27.4)	**.004**
Depression with medication	84 (15.7)	57 (15.9)	.93
MI	133 (24.9)	118 (33.0)	**.01**
Ventricular fibrillation or tachycardia	81 (15.1)	79 (22.1)	**.01**
Stroke	50 (9.3)	45 (12.6)	.13
Alcohol abuse	62 (11.6)	40 (11.2)	.85
Drug abuse	46 (8.6)	15 (4.2)	**.01**
Smoking history	(34.2)	(33.8)	.90
Cardiac procedures			
Prior PCI	91 (17.0)	77 (21.5)	.09
Prior CABG	69 (12.9)	84 (23.5)	**<.001**
Follow-up ablation	3 (0.6)	25 (7.0)	**<.001**
ICD type			**<.001**
ICD only	117 (21.9)	90 (25.1)	
Pacemaker only	8 (1.5)	17 (4.7)	
BiV pacer only	4 (0.7)	6 (1.7)	
BiV pacer with ICD	59 (11.0)	94 (26.3)	
Baseline anticoagulation	92 (17.3)	265 (74.4)	**<.001**
Baseline medications			
ACE inhibitor	357 (67.0)	206 (57.9)	**.01**
ARB	88 (16.5)	61 (17.1)	.81
ACE or ARB	442 (82.6)	267 (74.7)	**.01**
Beta blocker	503 (94.5)	340 (95.5)	.52
MRA	270 (50.7)	173 (48.6)	.55
90-day medications			
ACE inhibitor	278 (62.8)	164 (54.8)	.03
ARB	79 (17.8)	60 (20.1)	.44
ACE or ARB	355 (66.4)	224 (62.6)	.25
Beta blocker	421 (95.0)	285 (95.3)	.86
MRA	248 (56.0)	179 (60.1)	.27
NYHA class			**.03**
I	44 (8.3)	15 (4.2)	
II	277 (52.0)	169 (47.5)	
III	195 (36.6)	162 (45.5)	
IV	11 (2.1)	6 (1.7)	
Unknown	6 (1.1)	4 (1.1)	
Baseline laboratory values			
NT-proBNP (pg/mL)	4647 (7705)	4800 (5614)	.73
Serum creatinine (mg/dL)	1.39 (0.60)	1.55 (0.68)	**.001**
Serum sodium (mEq/L)	138.4 (3.7)	138.4 (3.6)	.98
Serum potassium (mEq/L)	4.4 (0.6)	4.4 (0.6)	.20
Serum BUN (mg/dL)	30.8 (23.1)	36.5 (24.8)	**.001**
NT-proBNP at 90 days (pg/mL)	3041 (4723)	3534 (4296)	.16
NT-proBNP ≥1000 pg/mL at 90 days (%)	81.5	89.4	**.002**
Ejection fraction (%)	23.1 (7.9)	25.9 (8.2)	**<.001**

a ACE, angiotensin-converting enzyme; AF, atrial fibrillation; ARB, angiotensin receptor blocker; BiV, biventricular; BMI, body mass index; BUN, blood urea nitrogen; CABG, coronary artery bypass graft; COPD, chronic obstructive pulmonary disease; CRT, cardiac resynchronization therapy; GUIDE-IT, Guiding Evidence-Based Therapy Using Biomarker-Intensified Treatment in Heart Failure trial; HFrEF, heart failure with reduced ejection fraction; ICD, implantable cardioverter-defibrillator; MI, myocardial infarction; MRA, mineralocorticoid receptor antagonist; NT-proBNP, N-terminal pro–B type natriuretic peptide; NYHA, New York Heart Association; PCI, percutaneous coronary intervention.

b To convert creatinine values to μmol/L, multiply by 88.4; to convert mEq/L values to mmol/L, multiply by 1; to convert NT-proBNP values to pmol/L, multiply by 0.1182; to convert BUN values to mmol/L, multiply by 0.357.

c Categorical variables are presented as number (percentage). Continuous variables are presented as mean (standard deviation). Boldface *P* values represent statistical significance.

However, AF patients had a significantly higher baseline ejection fraction and lower rates of prior drug abuse compared with those who did not have AF. Baseline NT-proBNP level was numerically higher but not significantly different between those with and those without AF. All other demographic and baseline comorbidity, laboratory, and medication data were not significantly different and can be found in Table 1.

In terms of GDMT, AF patients had a significantly higher percentage of target dose of beta blockers at baseline (*P*<.001) and 90 days (*P*=.01), but there were no significant differences in percentage of target dose of beta blockers at subsequent time points, including 6 months, 12 months, and 24 months (Table 2). There were no differences in percentage target dose of other GDMTs including angiotensin-converting enzyme inhibitors, angiotensin receptor blockers, and mineralocorticoid receptor antagonists at any time point (*P*>.05 all; Table 2). There were also no significant differences in the total number of visits between patients with AF and patients without AF (*P*=.51).

**Table 2:** Percentage of Patients Achieving Target Dose of GDMT at Baseline, 90 Days, 6 Months, 12 Months, and 24 Months Stratified by Baseline AF^a^^,^^b^

Variables	No AF (n=535), %	AF (n=358), %	*P* value
Baseline			
ACE inhibitor	39.8 (37.8)	41.8 (40.9)	.57
ARB	40.7 (27.8)	38.7 (30.4)	.69
Beta blocker	27.8 (22.9)	42.5 (61.4)	**<.001**
MRA	48.8 (25.4)	52.7 (27.9)	.14
90 days			
ACE inhibitor	49.9 (39.4)	50.7 (42.5)	.86
ARB	42.5 (29.4)	35.8 (32.8)	.55
Beta blocker	39.0 (35.4)	45.1 (29.6)	**.01**
MRA	55.5 (23.6)	55.1 (28.2)	.89
6 months			
ACE inhibitor	52.7 (39.2)	49.8 (39.4)	.49
ARB	49.8 (60.1)	41.9 (29.3)	.33
Beta blocker	43.5 (29.4)	46.4 (29.7)	.23
MRA	55.7 (32.3)	56.0 (31.5)	.93
12 months			
ACE inhibitor	57.9 (42.7)	50.1 (45.1)	.18
ARB	50.7 (47.7)	44.7 (30.9)	.44
Beta blocker	44.5 (30.4)	47.2 (30.9)	.34
MRA	59.8 (35.4)	55.2 (28.9)	.26
24 months			
ACE inhibitor	60.9 (46.3)	53.4 (44.1)	.39
ARB	49.5 (38.6)	42.9 (27.9)	.54
Beta blocker	51.3 (51.3)	42.0 (28.4)	.09
MRA	55.7 (32.3)	56.0 (31.5)	.36

a ACE, angiotensin-converting enzyme; AF, atrial fibrillation; ARB, angiotensin receptor blocker; GDMT, guideline-directed medical therapy; MRA, mineralocorticoid receptor antagonist.

b Values are reported as mean (standard deviation). Boldface *P* values represent statistical significance.

At 90 days, a higher proportion of patients with AF had an NT-proBNP concentration above 1000 pg/mL (89.4% vs 81.5%; *P*=.002). Patients with AF had a higher NT-proBNP concentration across the median 15 months of follow-up of the study (Figure). In addition, although NT-proBNP continued to improve gradually among patients without AF beyond 6 months with titration of GDMT, it began to increase beyond this time point for patients with AF (Figure).

**Figure:**
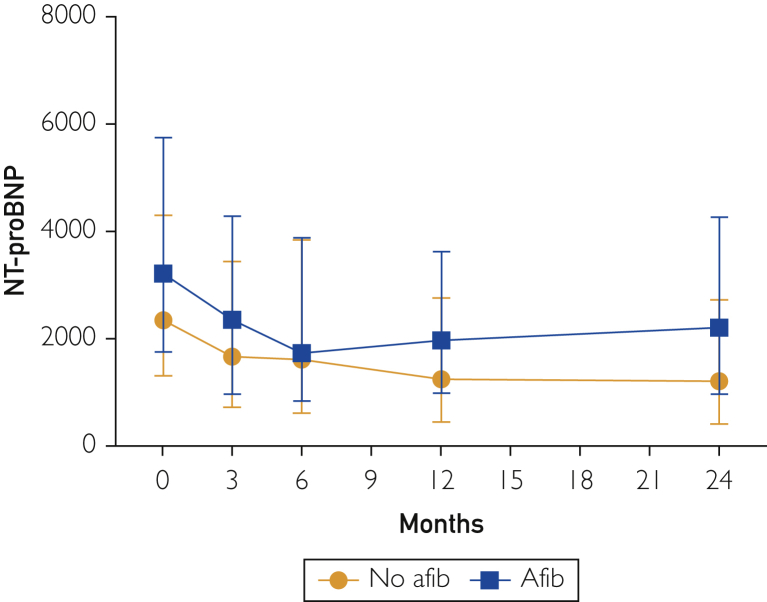
N-terminal pro–B type natriuretic peptide (NT-proBNP) levels trended over time by atrial fibrillation (Afib) status for patients with heart failure with reduced ejection fraction in the Guiding Evidence-Based Therapy Using Biomarker-Intensified Treatment in Heart Failure (GUIDE-IT) trial. Data are trended at 0, 3, 6, 12, and 24 months.

There were no significant differences between patients with and patients without AF in terms of unadjusted rates of adverse events during the study, severe adverse events during the study, all-cause death, or CV death (Supplemental Table 1, available online at http://mcpiqojournal.org). However, the rates of HF hospitalization or CV death, HF hospitalization, and all-cause hospitalization were significantly higher for those with AF compared with those without (*P*=.03 and *P*=.02, respectively; Supplemental Figure, available online at http://mcpiqojournal.org).

With use of Cox regression, the unadjusted hazards of HF hospitalization or CV death, HF hospitalization, and all-cause hospitalization were significantly higher for patients with AF compared with those without AF (Table 3). After adjustment, AF was associated with a statistically significant increase in the risk of HF hospitalization or CV death (hazard ratio, 1.28; 95% CI, 1.02 to 1.61; *P*=.04) and HF hospitalization (hazard ratio, 1.31; 95% CI, 1.02 to 1.68; *P*=.03). When stratified by NT-proBNP level of 1000 pg/mL, AF was associated with increased adjusted odds of HF hospitalization or CV death at baseline and HF hospitalization at both baseline and 90 days in the subgroup with an NT-proBNP level of 1000 pg/mL and higher but not in the subgroup with an NT-proBNP level below 1000 pg/mL at those time points (Table 4).

**Table 3:** Effect of AF on Odds of Adverse Events Among Patients With HFrEF in the GUIDE-IT Trial^a^^,^^b^

Outcomes	Unadjusted hazard ratio	*P* value	Adjusted hazard ratio^c^	*P* value
Death	1.33 (0.96-1.85)	.09	1.12 (0.79-1.59)	.53
CV death	1.26 (0.86-1.84)	.24	1.11 (0.74-1.66)	.61
HF hospitalization or CV death	1.29 (1.03-1.60)	**.03**	1.28 (1.02-1.61)	**.04**
HF hospitalization	1.28 (1.02-1.62)	**.04**	1.31 (1.02-1.68)	**.03**
Any hospitalization	1.22 (1.02-1.46)	**.03**	1.13 (0.93-1.37)	.21

a AF, atrial fibrillation; CV, cardiovascular; GUIDE-IT, Guiding Evidence-Based Therapy Using Biomarker-Intensified Treatment in Heart Failure trial; HF, heart failure; HFrEF, heart failure with reduced ejection fraction.

b Boldface *P* values represent statistical significance.

c Model adjusted for age, sex, diabetes, chronic kidney disease, prior myocardial infarction, prior stroke, ventricular arrhythmia, baseline NT-proBNP level, and baseline left ventricular ejection fraction.

**Table 4:** Subgroup Analysis Based on NT-proBNP Level of the Effect of AF on Adverse Events Using Cox Regression Models Among Patients With HFrEF in the GUIDE-IT Trial^a^^,^^b^^,^^c^

NT-proBNP level at baseline analysis				
Outcomes	Unadjusted hazard ratio	*P* value	Adjusted hazard ratio^d^	*P* value
Death				
NT-proBNP <1000 pg/mL	0.69 (0.14-3.34)	.65	0.27 (0.05-1.61)	.15
NT-proBNP ≥1000 pg/mL	1.30 (0.93-1.83)	.13	1.14 (0.79-1.64)	.49
CV death				
NT-proBNP <1000 pg/mL	—	—	—	—
NT-proBNP ≥1000 pg/mL	1.26 (0.86-1.85)	.24	1.13 (0.75-1.71)	.55
HF hospitalization or CV death				
NT-proBNP <1000 pg/mL	0.48 (0.16-1.41)	.18	0.56 (0.17-1.82)	.33
NT-proBNP ≥1000 pg/mL	1.30 (1.03-1.63)	**.02**	1.32 (1.04-1.67)	**.02**
HF hospitalization				
NT-proBNP <1000 pg/mL	0.54 (0.18-1.60)	.26	0.74 (0.22-2.50)	.63
NT-proBNP ≥1000 pg/mL	1.29 (1.02-1.65)	**.04**	1.34 (1.04-1.73)	**.03**
Any hospitalization				
NT-proBNP <1000 pg/mL	0.83 (0.44-1.56)	.57	0.87 (0.43-1.75)	.69
NT-proBNP ≥1000 pg/mL	1.22 (1.01-1.48)	**.04**	1.16 (0.95-1.42)	.14

NT-proBNP level at 90-day analysis				
90-day NT-proBNP	Unadjusted hazard ratio	*P* value	Adjusted hazard ratio^d^	*P* value
Death				
NT-proBNP <1000 pg/mL	1.19 (0.28-4.97)	.82	0.98 (0.19-4.99)	.98
NT-proBNP ≥1000 pg/mL	1.04 (0.67-1.62)	.85	0.94 (0.59-1.52)	.81
CV death				
NT-proBNP <1000 pg/mL	1.86 (0.26-13.18)	.54	0.50(0.04-6.06)	.58
NT-proBNP ≥1000 pg/mL	1.03 (0.63-1.69)	.92	0.93 (0.54-1.58)	.77
HF hospitalization or CV death				
NT-proBNP <1000 pg/mL	1.20 (0.55-2.66)	.65	1.15 (0.50-2.66)	.75
NT-proBNP ≥1000 pg/mL	1.21 (0.92-1.60)	.18	1.34 (1.00-1.81)	**.05**
HF hospitalization				
NT-proBNP <1000 pg/mL	1.25 (0.54-2.90)	.60	1.22 (0.50-2.95)	.67
NT-proBNP ≥1000 pg/mL	1.23 (0.92-1.64)	.17	1.40 (1.02-1.91)	**.04**
Any hospitalization				
NT-proBNP <1000 pg/mL	0.98 (0.56-1.71)	.95	0.92 (0.51-1.66)	.79
NT-proBNP ≥1000 pg/mL	1.13 (0.90-1.41)	.31	1.10 (0.87-1.40)	.44

a AF, atrial fibrillation; CV, cardiovascular; GUIDE-IT, Guiding Evidence-Based Therapy Using Biomarker-Intensified Treatment in Heart Failure trial; HF, heart failure; HFrEF, heart failure with reduced ejection fraction; NT-proBNP, N-terminal pro–B type natriuretic peptide.

b To convert NT-proBNP values to pmol/L, multiply by 0.1182.

c Boldface *P* values represent statistical significance.

d Model adjusted for age, sex, diabetes, chronic kidney disease, prior myocardial infarction, prior stroke, ventricular arrhythmia, baseline NT-proBNP level, and baseline left ventricular ejection fraction.

AF patients had no significant differences in KCCQ overall summary scores compared with those without AF (Supplemental Table 2, available online at http://mcpiqojournal.org).

## Discussion

The goal of our study was to evaluate differences in NT-proBNP levels and outcomes among patients with HFrEF with and without AF who were enrolled in the GUIDE-IT trial, an RCT designed to evaluate the efficacy of an NT-proBNP–guided HF treatment strategy. Whereas prior studies have reported that AF worsens outcomes in a community-based observational cohort of patients with HFrEF with variable management,^7^ this study found that this effect persists even in this contemporary trial cohort observed closely with frequent study-related clinic visits and having protocol-driven optimization of HF therapies.^1^ We found that among those with HFrEF, AF was associated with an increased risk of HF hospitalization or CV death and an increased risk of HF hospitalization alone; when stratified by NT-proBNP level of 1000 pg/mL, these findings persisted only among those with AF and NT-proBNP level of 1000 pg/mL and higher. In addition, we found higher NT-proBNP levels among patients with AF for all time points through 2 years of study follow-up and actually an increase in this biomarker after 6 months for those with AF compared with a decrease among those without AF despite ongoing efforts at HF medical therapy optimization. Use of beta blockers at target doses was actually higher among those with AF at baseline and 90 days, but otherwise there were no significant differences in percentage target medication titration for GDMT and no differences in total visit numbers between those with AF and those without AF. Our data therefore demonstrate a pathologic synergy between HFrEF and AF despite attempts for optimal HF medical treatment and biomarker evidence corroborating worse HF outcomes for those with AF.

Our analysis demonstrates a significant increase in resource utilization among patients with HFrEF and comorbid AF. Specifically, we found a significant increase in likelihood of HF hospitalization among patients with AF, controlling for differences in age, sex, diabetes, chronic kidney disease, prior myocardial infarction, prior stroke, ventricular arrhythmia, baseline NT-proBNP concentration, and baseline left ventricular ejection fraction. In addition, there was also a significant increase in the primary end point of GUIDE-IT, namely, a composite of time to first HF hospitalization or CV death for patients with AF. These findings are consistent with an analysis of patients in the Angiotensin-Neprilysin vs Enalapril in Heart Failure (PARADIGM-HF)^18^ and Aliskiren, Enalapril, or Aliskiren and Enalapril in Heart Failure (ATMOSPHERE)^19^ trials that found a higher risk of HF hospitalization, the primary composite end point of CV death or HF hospitalization, and stroke.^10^ Interestingly, we did not find a statistically significant difference in KCCQ score between those with and those without AF to explain these differences.

The reason for the observed increase in the primary composite end point of HF hospitalization or CV death and in HF hospitalization alone among those with HFrEF and AF in our study is unknown. AF and HF share many common risk factors and have consistently been shown to coexist in 15% to 35% of HF patients.^2^, ^3^, ^4^, ^5^, ^6^ Whereas outcomes have been shown to be worse among those with HF and AF in the past,^3^^,^^20^, ^21^, ^22^ our data suggest that this pathologic relationship persists despite considerable advances in HF treatment and even when patients are frequently observed with up-titration of GDMT. Furthermore, we found that there were no differences in the percentage target doses of GDMT achieved, except for a higher target dose of beta blockers achieved among patients with AF at baseline and 90 days. Atrial fibrillation is associated with tachycardia and irregular conduction that can lead to a loss of atrial systole, decreased diastolic filling interval, decreased cardiac output, increased end-diastolic pressures, and increased neurohormonal activation.^23^ It has also been reported that AF seems to negate the benefits of beta blockers among those with HFrEF.^24^ These factors all conspire to increase the risk of worsening HF in patients with AF. Prior studies, including the Catheter Ablation for Atrial Fibrillation with Heart Failure (CASTLE-AF) trial, have reported an improvement in HF hospitalization and functional status in patients with HFrEF and AF undergoing catheter ablation compared with medical therapy alone.^25^ Whereas our data do not directly assess the role of AF rhythm control, they do show that AF has pathologic consequences even with the most contemporary and intensive medical therapy, suggesting that AF rhythm control may offer an advantage even under those circumstances.

Despite a premature termination of the GUIDE-IT trial because of futility, there was still a median follow-up of 15 months for the patients included.^1^ Kaplan-Meier curves comparing survival free of the primary end point for the 2 groups continue to diverge over time, with survival worse in the AF group (Supplemental Figure). We also observed a separation in the quantitative NT-proBNP curves, with higher NT-proBNP levels in the AF population, which was maintained over time. Although patients without AF had a decrease in NT-proBNP level during up-titration of GDMT, those with AF had a different trajectory, with an NT-proBNP nadir at approximately 6 months followed by gradual increase. To our knowledge, this longitudinal trend of NT-proBNP levels in patients with HFrEF and AF has not been described. Although we cannot definitely determine causation, these data may suggest a putative mechanism by which AF contributes to worse outcomes in HFrEF over time. Notably, we found that among those with AF, the adjusted odds of HF hospitalization or CV death and HF hospitalization were higher only with an NT-proBNP concentration of 1000 pg/mL and higher, suggesting that this biomarker may be particularly prognostic among those with HFrEF and AF.

There are multiple limitations to this study. As a secondary analysis of an RCT, we cannot completely eliminate the possibility that residual confounding contributed to the observed differences in outcomes between those with AF and those without AF. This paper is subject to the limitations inherent to a cohort study, and causation cannot be inferred from this study. However, we adjusted our analysis for several sociodemographic and medical characteristics, and our findings were consistent for multiple unadjusted and adjusted analyses. The trial data did not include longitudinal information on the detailed atrial arrhythmia history of patients. More detail about AF or atrial flutter chronicity and treatment including antiarrhythmic drug use, detailed oral anticoagulation data, or prior ablation procedures may augment our understanding of the complex interaction between HFrEF and AF. The lack of data on medical management of AF therefore remains an important limitation to this study. Finally, it is possible that findings from the GUIDE-IT trial may not be generalizable to a systolic HF population outside of a clinical trial setting, although the effect of AF would be expected to be even more profound in patients with less frequent follow-up and poorer HF medical treatment.

## Conclusion

Our analysis demonstrates the association between AF and worse outcomes in HFrEF among a contemporary population of HF patients being frequently monitored and receiving intensive HF medical management. We showed that AF was associated with an increased risk of HF hospitalization or CV death and an increased risk of HF hospitalization alone. In addition, we found higher NT-proBNP levels among patients with AF through 2 years of study follow-up and actually an increase in this biomarker after 6 months for those with AF despite ongoing efforts at HF medical therapy optimization. Our data demonstrate the pathologic relationship between HFrEF and AF despite optimal HF medical treatment and biomarker evidence corroborating worse HF outcomes for those with AF.
