# Supplementary material for: Effects of Atrial Fibrillation on Heart Failure Outcomes and NT-proBNP Levels in the GUIDE-IT Trial
Source: Mayo Clin Proc Innov Qual Outcomes. 2021 Apr 8;5(2):447–55. doi: 10.1016/j.mayocpiqo.2021.02.005 (PMC8105522; doi:10.1016/j.mayocpiqo.2021.02.005)
Supplement: Supplemental Tables 1 and 2, and Figure 1 [file mmc1.docx]

**Supplemental Materials**

**Figure Legends**

**Supplemental Materials Figure 1.** Kaplan-Meier for HF Hospitalization or CV Death - Kaplan-Meier curves for proportion of HF hospitalization or CV death. These curves compare those with HFrEF with and without AF in the GUIDE-IT trial with a log rank test.

**Supplemental Materials Table 1.** Unadjusted rates of adverse events by AF status over entirety of the GUIDE-IT trial.

| **Variables** | **No AF**  **(N=535)** | **AF**  **(N=358)** | **P-Value** |
| --- | --- | --- | --- |
| Adverse Event | 147 (27.4) | 117 (32.6) | 0.10 |
| Serious Adverse Event | 44 (8.3) | 32 (9.8) | 0.42 |
| Death | 75 (14.1) | 66 (18.5) | 0.07 |
| CV Death | 59 (11.1) | 49 (13.8) | 0.23 |
| HF Hospitalization or CV Death | 179 (33.4) | 148 (41.3) | **0.02** |
| HF Hospitalization | 210 (39.2) | 130 (36.2) | **0.03** |
| Any Hospitalization | 264 (49.3) | 206 (57.6) | **0.02** |
| Hospitalized/CV Death by day 30 | 40 (7.5) | 27 (7.6) | 0.97 |
| Hospitalized/CV Death by day 90 | 72 (13.5) | 50 (14.0) | 0.82 |

**Supplemental Materials Table 2.** KCCQ overall summary scores by AF status through 2 years of follow-up.

| **Variables** | **No AF** | **AF** | **P-Value** |
| --- | --- | --- | --- |
| Median Overall KCCQ Score (IQR) |  |  |  |
| Baseline | 59.3 [41.0-75.6] | 59.5 [40.9-74.3] | 0.759 |
| 3 Months | 74.0 [49.6-87.0] | 75.0 [56.8-90.0] | 0.196 |
| 6 Months | 76.1 [57.9-89.0] | 73.6 [54.9-88.5] | 0.682 |
| 12 Months | 74.5 [52.8-89.8] | 74.8 [52.3-90.3] | 0.930 |
| 24 Months | 76.4 [52.9-90.7] | 77.9 [57.3-91.4] | 0.618 |

**Supplemental Materials Figure 1.** **Kaplan-Meier for HF Hospitalization or CV Death** - Kaplan-Meier curves for proportion of HF hospitalization or CV death. These curves compare those with HFrEF with and without AF in the GUIDE-IT trial with a log rank test.


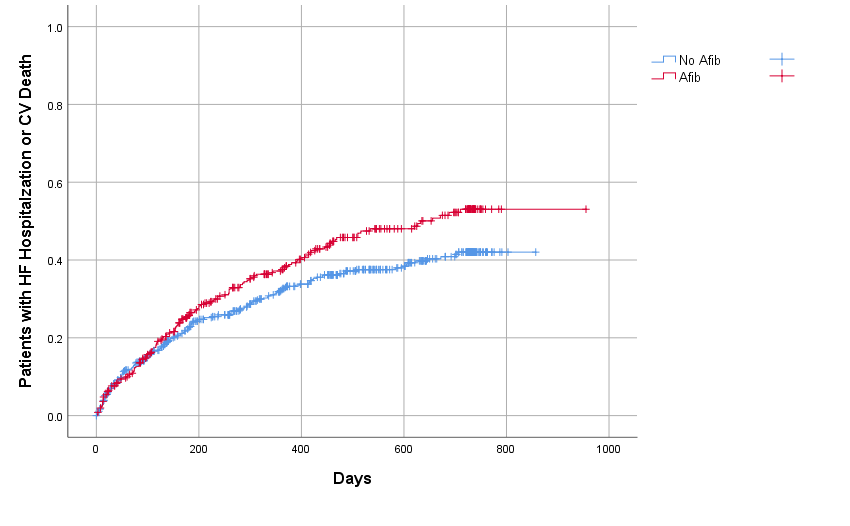


Log Rank Test: p=0.02
